# Endoscopic ultrasound-guided choledochoduodenostomy using an innovatively shaped self-expandable metal stent to prevent stent migration

**DOI:** 10.1055/a-2439-3793

**Published:** 2024-11-08

**Authors:** Takeshi Ogura, Takafumi Kanadani, Nobuhiro Hattori, Kimi Bessho, Hiroki Nishikawa

**Affiliations:** 1 38588 Endoscopy Center, Osaka Medical and Pharmaceutical University Hospital, Takatsuki, Japan; 2 13010 2nd Department of Internal Medicine, Osaka Medical and Pharmaceutical University, Takatsuki, Japan

Endoscopic ultrasound-guided choledochoduodenostomy (EUS-CDS) is now widely performed, not only as an alternative biliary drainage technique when endoscopic retrograde cholangiopancreatography (ERCP) fails, but also as primary drainage for unresectable malignant distal biliary obstruction 1 2 3 . The adverse event of stent migration sometimes occurs. In addition, EUS-CDS using a lumen-apposing metal stent (LAMS) has been reported, but if the common bile duct is not very dilated, this technique might be challenging, and misdeployment might occur, especially in nonexpert hands.

A novel tapered self-expandable metal stent (T-SEMS) (K-papilla biliary stent; S&G Biotech, Seoul, Korea) is now available in Japan ( Fig. 1 ). The main body of the T-SEMS has a diameter of 10 mm, but the stent is tapered toward the ampulla of Vater area, and at the ampulla of Vater the body diameter of the T-SEMS is 8 mm. With this unique design, an expanding force works toward the hepatic hilar region, and stent dislocation can be prevented. In addition, a wide flare (16 mm in diameter) at the duodenal end prevents stent migration. Furthermore, the absence of flare at the distal end prevents stent-induced ductal change. This stent might be useful to prevent stent dislocation and migration not only related to ERCP, but also to EUS-CDS. Herein, technical tips for EUS-CDS using T-SEMS are provided.

**Fig. 1:**
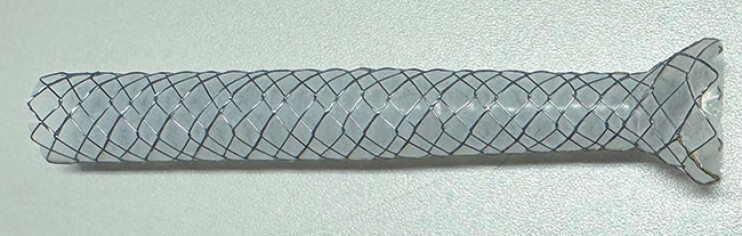
A novel tapered self-expandable metal stent for biliary drainage (K-papilla biliary stent; S&G Biotech, Seoul, Korea).

An 81-year-old man was admitted to our hospital because of obstructive jaundice caused by cancer of the head of the pancreas. Because of failed biliary cannulation due to tumor invasion into the ampulla of Vater, EUS-CDS was attempted. First, the common bile duct was punctured using a 19-G needle, and the contrast medium was injected ( Fig. 2 ). After a 0.025-inch guidewire was deployed within the biliary tract ( Fig. 3 ), the common bile duct and duodenal wall were dilated using a 4-mm balloon catheter. Then, the stent delivery system of the T-SEMS was successfully inserted and deployed ( Fig. 4 ), without any adverse events. Although the proximal site of the stent was short, to prevent duodenal mucosal injury, no stent migration was observed after 7 days ( Fig. 5 , Video 1 ).

**Fig. 2:**
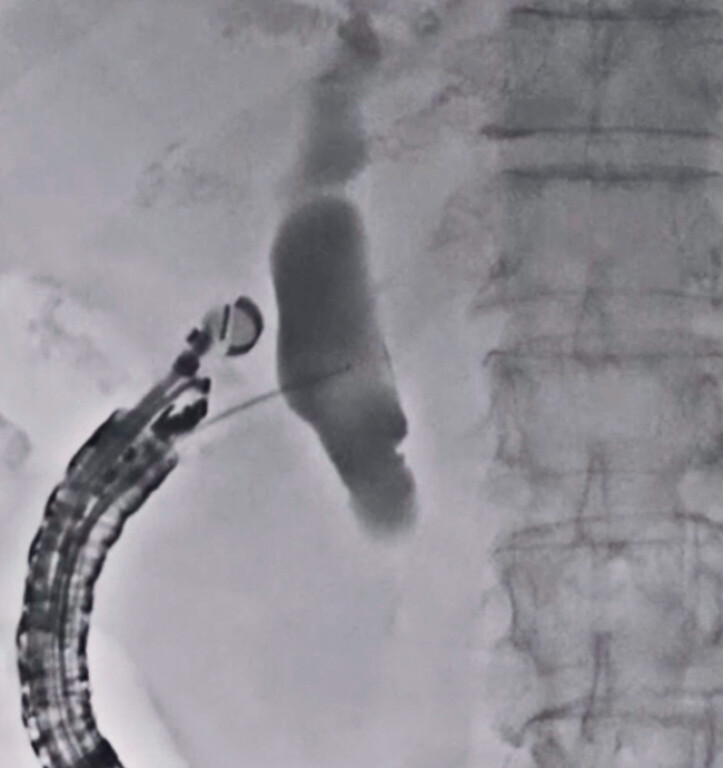
At endoscopic ultrasound-guided choledochoduodenostomy (EUS-CDS), the common bile duct is punctured using a 19-G needle and the contrast medium is injected.

**Fig. 3:**
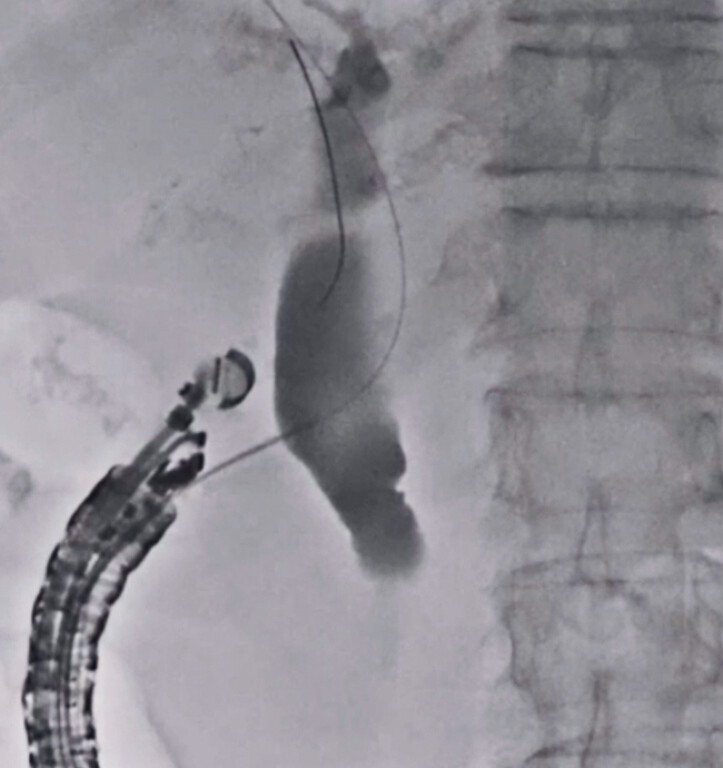
A 0.025-inch guidewire is deployed within the biliary tract.

**Fig. 4:**
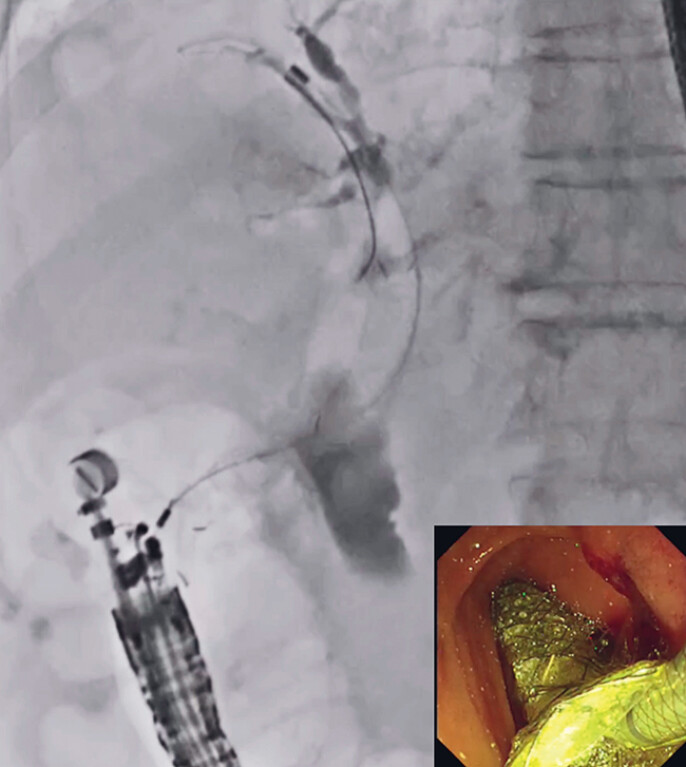
The stent delivery system of a novel tapered self-expandable metal stent is successfully inserted and deployed.

**Fig. 5:**
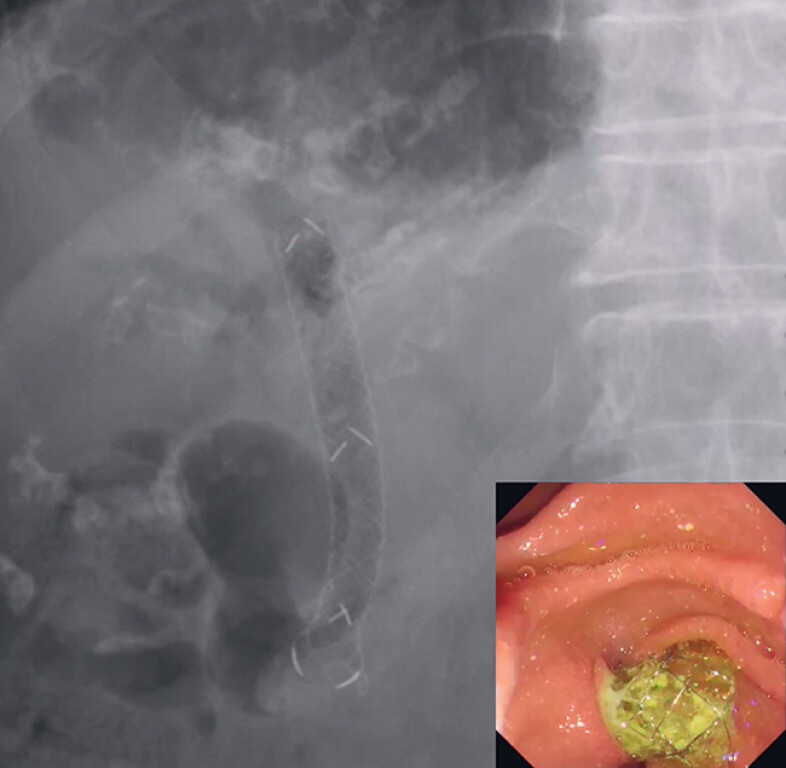
No stent migration is observed after 7 days.

Endoscopic ultrasound-guided choledochoduodenostomy is successfully performed using a novel tapered self-expandable metal stent.Video 1

In conclusion, the T-SEMS might be useful to prevent stent dislocation and migration not only related to ERCP, but also to EUS-CDS, although a comparison study is needed.

Endoscopy_UCTN_Code_TTT_1AS_2AH

## Notes

Endoscopy E-Videos https://eref.thieme.de/e-videos
*E-Videos* is an open access online section of the journal *Endoscopy* , reporting on interesting cases and new techniques in gastroenterological endoscopy. All papers include a high-quality video and are published with a Creative Commons CC-BY license. Endoscopy E-Videos qualify for HINARI discounts and waivers and eligibility is automatically checked during the submission process. We grant 100% waivers to articles whose corresponding authors are based in Group A countries and 50% waivers to those who are based in Group B countries as classified by Research4Life (see: https://www.research4life.org/access/eligibility/ ). This section has its own submission website at https://mc.manuscriptcentral.com/e-videos .
